# Seeking the aim – perspectives of asylum seekers, nurses, and authorities on the objectives of the asylum seekers’ initial health assessment: a qualitative study

**DOI:** 10.1186/s12913-024-11531-w

**Published:** 2024-09-27

**Authors:** K-L Mustonen, M. Ismail, T. Mäki-Opas, A.E. Castaneda, J. Kauhanen, S. Atkins, N. Skogberg

**Affiliations:** 1 Finnish Institute for Health and Welfare, Helsinki, Finland; 2 University of Eastern Finland, Kuopio, Finland; 3 Wellbeing Services County, Wellbeing Services Research Center, North Savo, Kuopio, Finland; 4 University of Tampere, Tampere, Finland

**Keywords:** Effectiveness, Asylum seeker, Health assessment, Vulnerable groups

## Abstract

**Background:**

A voluntary and free initial health assessment is offered to all asylum seekers upon arrival in Finland. The central aim of this initial health assessment is early identification of service needs. There is, however, limited information on how effective the initial assessment is in fulfilling its aims. This study explores the viewpoints of asylum seekers, reception centre nurses, and health authorities regarding the objectives of the initial health assessment. It serves as a starting point for effectiveness research, where effectiveness is defined as the achievement of intended aims.

**Methods:**

This qualitative descriptive study is based on 31 semi-structured individual interviews (13 asylum seekers, 14 nurses, and four asylum health authorities) conducted in January and February 2019. Reflective thematic analysis was employed for data analysis, involving initial separate analyses for each group, followed by an assessment of differences and similarities between the groups.

**Results:**

The importance of a comprehensive initial health assessment and preventing infections was emphasized by all groups. The main differences were views on service needs assessment in relation to persons in vulnerable situation and information provision. All groups described both individual and public health perspectives.

**Conclusions:**

This study provides valuable insights for developing a more effective assessment. Asylum seekers require comprehensive health assessment and details about their rights. To address these needs, it is crucial to update reception centre nurses’ practices. Additionally, authorities responsible for planning and guiding services should refine their instructions concerning the information provided to asylum seekers and persons in vulnerable situations. The findings of this study can be used to enhance information provision and develop targeted training programs for nurses, as well as to evaluate the achievement of established aims.

**Supplementary Information:**

The online version contains supplementary material available at 10.1186/s12913-024-11531-w.

## Background

The impact of migration on a person’s health is influenced by various factors including their parents’ history, biological factors such as genes and sex, reasons for migration, and the individual’s environment and context in different phases of life [1]. Among all migrants, prior health risks, the hazards of the journey, and experiences of uncertainty and othering in the receiving country, place forced migrants in a particularly vulnerable position [2–4]. Although asylum seekers are still in the process of applying for international protection and refugees have already received a positive decision, these groups share similarities and are both referred to in the background section of this study [5].

Asylum seekers’ health is further characterized by political and structural impediments to accessing medical treatment. Directive 2013/33/EU, known as the Recast Reception Conditions Directive, sets basic criteria for asylum seekers’ access to healthcare throughout the asylum process [6]. It stipulates that the European Union (EU) Member States guarantee that asylum seekers receive, at a minimum, emergency care and necessary treatment for physical and severe mental health conditions. Nevertheless, the scope of healthcare services available to asylum seekers is restricted by national laws, resulting in Member State variations.

Asylum seekers’ initial health assessments (IHAs) often target both individual and public health objectives [7]. At the individual level, the general aim of the IHAs is to identify instances of poor health and promote overall well-being. At the whole population level, IHA serves to ensure the safety and health of residents within the receiving country. Concurrently, these endeavours contribute to broader societal advantages in terms of integration and the economy [8]. Examining initial health assessments is crucial for enhancing the early identification of service needs during the initial stages of the asylum process and the service pathway.

### Asylum seekers’ healthcare in Finland

The number of asylum seekers in Finland has typically fluctuated between 1,500 and 6,000 applicants per year during the 2000s [9]. However, in 2015, an exceptional amount of applications was witnessed reaching 32,477. Notably, due to the Russian aggression on Ukraine, a substantial peak occurred in 2022, with 47,302 Ukrainians seeking protection in Finland. Previously, consistent numbers of Turks, Iraqis, Russians, Afghanis, and Somalis applied for protection in Finland.

The Finnish Immigration Service, operating under the Ministry of the Interior, monitors processing asylum appeals and delivering reception services [10]. The Finnish Immigration Service collaborates with medical experts from the Finnish Institute for Health and Welfare (THL) in organizing and directing asylum seekers’ healthcare.

The Act on the Reception of Persons Applying for International Protection and on the Identification of and Assistance to Victims of Trafficking in Human Beings (746/2011) outlines asylum seekers’ services [11]. After seeking asylum in Finland, the applicant is registered in a reception centre. The reception centre is responsible for accommodation and social and healthcare services. An asylum seeker can live either in the reception centre, other housing provided by the reception centre, or in private accommodation. During early stages of arrival, asylum seekers often live in so-called transit reception centres, from where they are usually transferred to longer-term housing to await for their asylum decision.

Throughout the asylum process, healthcare services are provided by the reception centre. The services are arranged together with the public sector and/or purchased from the private service providers. An adult asylum seeker is entitled to urgent and essential care. Urgent care includes immediate medical and dental treatment, while essential care covers diagnostics, disease identification, and treatment deemed necessary by healthcare professionals. Healthcare professionals often perceive definition of necessary care ambiguous. The two guiding principles that are often used are that a long-term illness would worsen without treatment, and that the assessment is based on medical, rather than cost consideration. Minor asylum seekers are entitled to the same healthcare services as permanent residents in Finland, including preventive services. In accordance with Sect. 6 specific requirements arising from asylum seekers’ vulnerable situation merit consideration, including factors such as age or physical and mental condition. It emphasizes the timely and individual identification and subsequent needs shortly after case initiation. Special needs are considered throughout the entire asylum process and for that period of time that victims of human trafficking are provided with dedicated services.

In Finland, every asylum seeker is offered a voluntary IHA upon arrival. The IHA is conducted by a reception centre nurse [10]. Individuals in vulnerable situations are additionally offered a doctor’s appointment. The IHA includes an interview, a health examination (HE), screening for communicable diseases, vaccinations, and an information session about entitlement to healthcare services. The interview covers background information, growth and development, illnesses and medical procedures, current health status, mental health, health habits, sexual and reproductive health, risk factors, and medications. The health examination consists of a skin and oral health examination, and measurements of blood pressure, body temperature, height, and weight. Screening for tuberculosis, HIV, hepatitis B, syphilis, and intestinal parasites and vaccinations are offered based on individual assessment. Typically, an adult asylum seeker’s chest X-ray and immunization status for diphtheria, polio, and measles are reviewed. Further care is provided based on the individual assessment.

To our knowledge, this study is one of the first to examine the primary aims of the asylum seekers’ IHA simultaneously from multiple perspectives: asylum seekers, reception centre nurses, and health authorities. Previous studies have focused on each group separately, revealing that asylum seekers may perceive the IHA as unpleasant and have insufficient information [12, 13]. Furthermore, research on effective refugee health nursing practices remains limited [14]. From the providers’ perspective, there is a recognized need to address the specific needs of asylum seekers, standardize procedures, as well as to assess the effectiveness of health examinations [15].

### Chain of effectiveness (IOOE) framework

Effectiveness can be defined as the successful achievement of intended aims [16]. These objectives serve as the foundation for guiding actions, determining the necessary resources and expertise (input), and shaping the practices that are implemented (output). In a comprehensive effectiveness analysis, a continuous focus is placed on monitoring both the objectives and their corresponding outcomes across multiple levels: the micro level (patient outcomes), the meso level (professional practices), and the macro level (societal impacts).

Furthermore, these levels can be employed to analyse power relations [17]. In this context, the micro level refers to the interactions between a nurse and an asylum seeker, where the dynamics of giving and receiving care can be significantly influenced by the approach to essential care. Nurses, in turn, are subject to the rules and practices of their workplace, as well as to directives from authorities which is referred to as the meso level. These factors are reflected in the behaviour and interactions of nurses as experienced by asylum seekers. The macro level encompasses national and international regulations. It is important to note that these regulations are implemented through individuals and interpersonal relationships.

### The aim of the study

The aim of this study is to describe the aim/s of the IHA from the perspective of asylum seekers, nurses, and authorities.

The specific research questions were as follows:

- How do asylum seekers, reception centre nurses, and asylum health authorities describe the aims of asylum seekers’ IHA?
- What similarities and differences can be observed in the responses of these different groups?

### Situated knowledge and research positionalities

Marian Ismail: I am writing from the perspective of an individual with a refugee background, serving in the capacities of both a professional nurse and a project worker on this topic. I acknowledge my roles as a refugee and healthcare worker, aiming to share a more in-depth understanding with participants.

Katri-Leena Mustonen: I am writing from the perspective of a former reception centre nurse and a project worker in connection with this topic. I consider my previous knowledge and my relationship with the field and professionals.

Natalia Skogberg: I am writing from the perspective of a health authority and manager of the project on development of the IHA protocol for asylum seekers, as well as a senior expert on the topic of migration and health.

## Methods

### Design

Qualitative Description (QD) was employed in the study. This method utilizes a combination of techniques to seek answers that are particularly relevant to practitioners and policymakers, making it well suited for needs assessment and research involving vulnerable groups [18, 19]. In this study purposive sampling and thematic interviews and analysis were used, explained in more detail in the following. The QD approach was deemed most appropriate because it does not necessitate complex data transformation and offers a comprehensive summary in a language that facilitates participants’ collaboration in the development process.

### Characteristics of participants

The participants were selected using purposive sampling, a strategy frequently employed in Qualitative Description (QD) studies [20]. Using this strategy, by defining specific criteria, participants who can provide in-depth information on a particular issue were selected. The inclusion criteria were as follows: 1) for asylum seekers (n = 13), a) newly arrived adult asylum seekers, and b) those who had participated in the IHA conducted in the reception centre, 2) for nurses (n = 14), a) employed in a reception centre, and b) involved in conducting IHAs,  and 3) for authorities (n = 4), a) Finnish immigration services experts responsible for provision and supervision of the health services for asylum seekers and b) other experts supporting organisation of asylum seekers’ health services.

The total number of asylum seekers participating in the study was thirteen: seven males and six females. The appropriate sample size was assessed throughout the study. Sampling was concluded once data saturation was reached, at which point additional data no longer provided novel insights [21], p. 59). The ages of the interviewees ranged from twenty to fifty-two years, a deliberate variation that was considered suitable for capturing a broad spectrum of the population under investigation. These individuals represent major regions of origin that sought asylum in Finland in 2018. The researcher (M.I.) established a collaborative relationship with reception center nurses prior to commencing the study while working on the Developing the health examination protocol for asylum seekers in Finland: A national development project (TERTTU). Information about the proposed research was provided to the reception center nurses to facilitate the recruitment of potential participants, who were then invited to consider participating voluntarily.

Subsequently, the participants were individually met by the reception center nurse, who informed them about the study and sought voluntary participation. The interviews were conducted in the native language of each participant, facilitated by a professional interpreter recruited from a private interpretive company, who translated the responses into Finnish for the researcher via telephone*.*

All nurses (n = 97) working in Finnish reception centres were invited to participate through emails and by promoting the study during their meetings. Due to the limited number of participants, detailed background information was not collected. However, based on the interviewer’s (K-L.M.) observations, there was good diversity among the participants in terms of location (covering the entire Finland), work experience in reception centres (less than one year n = 2; less than five years n = 8; more than five years n = 3), and education (including psychiatric nurses, midwives, paramedics, registered nurses, and public health nurses). During the study period, IHAs were mostly conducted in transit reception centres, allowing us to reach most of the nurses actively performing IHAs.

The health authorities responsible for provision and supervision of asylum-related healthcare in Finland consist of individuals employed by the Finnish Immigration Service and the Finnish Institute for Health and Welfare (THL). The group size is limited to a few professionals. Individuals within these organizations were targeted for inclusion in the study. Invitations to participate in the research were extended during collaborative meetings. Ultimately, four participants from this group participated in interviews conducted by N.S. as part of the study.

### Data collection

Semi-structured interviews were conducted from January 2019 to February 2019 by one interviewer per group (M.I., K-L.M., & N.S.) with a background similar to that of the group. All the data were recorded and transcribed verbatim, with lengths varying from 29 to 98 min.

A distinct qualitative interview guide was created for each group (see APPENDIX 1, 2 and 3). It included four themes with several sub questions: 1) the IHA as a phenomenon, 2) good practices in the IHA, 3) challenges in the IHA, and 4) development needs and proposals for the new IHA protocol. This article specifically focuses on the first theme. The interview framework was adapted and modified from previous research and similar development projects.

### Data analysis

The inductive data analyses were carried out independently by two researchers, M.I. (for asylum seekers) and K-L.M. (for nurses and authorities). Initially, data pertaining to the aims of the IHA were extracted from the extensive dataset. Both researchers applied [22] thematic analysis. Initially, all transcripts were meticulously read and open-coded to identify recurring themes. Quotations were organized based on a preliminary thematic map that included shortened phrases, codes, subthemes, themes, and main themes. The codes were standardized, and the overall coherence of the map was scrutinized. Subsequently, quotations were grouped according to themes, and the coherence of these themes was confirmed. Finally, maps from all participant groups were compared, and differences and similarities were examined. This method was chosen for its accessibility to a junior researcher, its theoretical flexibility, and its suitability for engaging participants as collaborators.

### Ethical considerations

Ethical approval from the regional ethics committee was received (3306/2017). All participants were informed of their rights, agreed on recording the interviews and signed a written consent form. The principles of the Declaration of Helsinki were respected during the study.

Special attention was paid to the informed consent of asylum seekers. Having prior experience working with asylum seekers the interviewer understood that some participants might have undergone traumatic events. Therefore, compartmentalizing their memories to avoid distress and re-traumatization was important. To address potential language barriers, professionally qualified interpreters were employed to translate the participants’ native languages. To maintain confidentiality, the collected data were pseudonymized, and numerical codes were utilized in this paper to protect the anonymity of the participants. Furthermore, all personally identifiable information was removed, and participants’ countries of origin were categorized by continents to prevent the stigmatization of interviewees due to the sensitive nature of the discussions and to avoid potential identification. In the transcription process, each participant received a code; however, these codes were not utilized in the extracts to ensure confidentiality.

## Findings

**Fig. 1:**
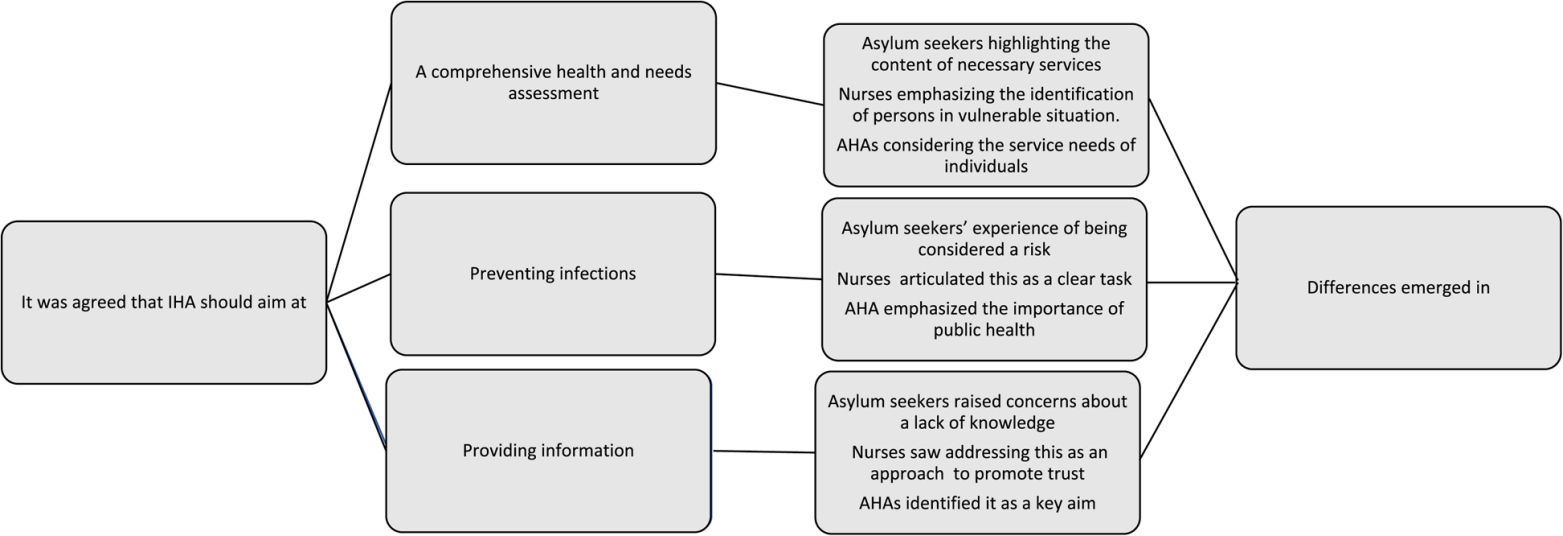
Summary of the findings

The findings of the study were organized into three main themes: a comprehensive health and needs assessment, preventing infections, and providing information. Figure 1 summarizes the findings. The data patterns are illustrated through the presentation of themes, categories, and subcategories (see Additional file 1. Table 1 A summary of the findings).

### Comprehensive health and needs assessment

Asylum seekers had varied opinions on the comprehensiveness of the assessment. Especially those with chronic illnesses or non-urgent conditions were dissatisfied with the content of the assessment. Participants highlighted structural challenges in implementing the IHA, citing limited content and time frame as compromising its effectiveness. They emphasized the importance of a holistic approach to the IHA and other initiatives to address the health and care needs of new asylum seekers. Interviewees stressed the necessity for additional psychological diagnostics and care for traumatized asylum seekers. They recommended psychologists assess and guide vulnerable asylum seekers to immediate care if required, providing discussion sessions in their own space and time. Moreover, they proposed further guidance on processing and coping with trauma.

“In my opinion, an initial health assessment should be added to an interview with a psychologist. A person may have experienced different things, e.g., someone could have been raped or something else. A person may not be able to tell all about what has happened during the health check-up, but a psychologist who is a professional he/she might tell, or the psychologist can get out more of this type of people who are vulnerable” (Female, Middle East/North Africa).

Some nurses emphasized evaluating health status as the primary goal of the IHA. This assessment includes an individual’s health history, current health conditions requiring immediate attention, necessary treatments, and medication needs. However, there was a desire to adopt a client-oriented approach, enabling clients to express their coping mechanisms. Prioritizing emerging needs and ensuring follow-up care were identified as crucial aspects, with a comprehensive evaluation of health status recognized as foundational for future interventions.

For some nurses, identifying those in vulnerable situation was deemed the most important aim of the IHA. They highlighted the importance of recognizing patterns, assessing functional capacity, and uncovering the reasons behind seeking asylum and any experienced violence. Building a trusting relationship, particularly in identifying vulnerable individuals, was emphasized, requiring consistent interaction and time investment. Proactive exploration of potential health issues related to trauma, abuse, torture, and sexual violence was considered essential. Following identification, nurses considered multi-professional assistance for those in vulnerable situations and the possibility of uncovering human trafficking cases through social workers. Records were suggested to include headings related to vulnerability and perceived violence for documentation purposes. Interviewees note that many asylum seekers may demonstrate traumatic stress reactions, which may become apparent later in the process. Additionally, participants highlighted that even if vulnerability is acknowledged, immediate treatment may not be required. Nurses emphasized the need for supplementary training and tools for more effective identification of vulnerability.

*“It can be the narrative shared by the client, the unspoken nuances within the story, or even certain behaviours exhibited in specific situations or an appearance. There seem to be numerous contributing factors, yet the overall impression comes from somewhere. I make a conscious effort to allocate more time and space, providing the client with the opportunity to open up.”*

According to the asylum health authorities (AHAs), central to the first theme is that a comprehensive IHA marks the beginning of health services in reception facilities, and it is a legal requirement that must be completed within a specified time frame. Based on the observations, the service system is oriented towards providing the essential treatment or services. They further stated that the primary function of the IHA and subsequent services is to assess an individual’s physical and mental condition and identify any resulting special needs. From the perspective of the healthcare provider, addressing these special needs and organizing services for them was perceived to be more practical than defining a group of people in a vulnerable situation. AHAs stated that it allows the client to define their own needs. However, they raised an ethical question whether the essential services are available.

*“In the initial health assessment, it is essential to comprehensively assess not only a person’s physical well-being but also their psychological well-being and identify any support needs.”*

### Preventing infections

Asylum seekers conveyed that the IHA encompasses a blood test, chest X-ray, HE, and vaccinations. They articulated that the IHA effectively communicates the necessity of these procedures, emphasizing its purpose to control infectious diseases and implement preventive measures that prove advantageous for both individuals and society as a whole. Nearly all asylum seeker participants conveyed the significance of the IHA for both individual well-being and the broader societal context. They emphasized that the primary purpose of the HE was the identification of infectious diseases and the implementation of preventive measures. Only one participant expressed uncertainty regarding the rationale behind health examinations. Additionally, asylum seekers articulated their demand for healthcare services beyond acute care, particularly as some participants grappled with long-term illnesses. Approximately half of the asylum seeker participants believed that the IHA aimed to determine the presence of infectious diseases, considering this information crucial for their well-being and expressing trust that authorities would provide assistance if an infection were detected.

“I believe that the aim of the initial health assessment is to determine whether we have infectious diseases that could be infected to other people. I mean, if this examination shows that a person has illness, first they inform the person then they will guide them on how to proceed.” (Female, Russia/former Soviet Union).

Nurses stated that the primary focus of their work revolves around identifying infectious diseases. Furthermore, they expressed that infection prevention instructions play a crucial role as an important tool in this context. The emphasis during the IHA lies in screening tests and vaccinations. The nurse interviewees stated that other health issues and examinations may be addressed over an extended timeframe. Screenings following the country-of-origin protocol were described as a clearly defined component of the IHA. However, some nurses expressed concerns that asking questions related to transit countries blurred the lines between the roles of the police and nurses. Participants emphasized that beyond identification and treatment, the overarching aim of the IHA is to prevent the spread of infectious diseases. Nurses address clients’ difficulties in articulating symptoms, diseases, and conditions as a notable challenge, hindering the early detection of infectious diseases. The interviewees believed that nurses’ role is crucial in explaining the advantages of vaccinations and screening tests to clients. Furthermore, they stated that recording and transferring the results of screening tests and vaccinations are considered essential components of the process.

*“Let us ensure rapid access to treatment for those who genuinely need it or may be carriers of conditions such as diseases that could pose broader risks. In other words, early detection of infectious diseases and immediate initiation of appropriate treatment are crucial aspects in addressing such concerns.”*

AHAs highlighted that preventing infections encompasses not only the individual but also protecting the public against communicable diseases. They stated that symptoms and infectious diseases should be identified during the IHA. This includes receiving initial health information either in a group or individually, meeting with a nurse, vaccinations, laboratory tests, chest X-ray, a doctor’s check-up, and addressing symptoms within the specified timeframe. They further state that it is a statutory and comprehensive assessment to determine the need for urgent and necessary treatment. AHAs noted that the Finnish Institute for Health and Welfare and the Ministry of Social Affairs and Health are involved in the IHA from an infectious disease perspective.

*“In these screening examinations, which are part of the initial health assessment, the individual’s needs are on display. Many of these screenings also encompass the aspect of protecting civilians/the public against communicable diseases. The primary aim, of course, is to prevent the spread of infectious diseases within our population, particularly since the reception centre creates favourable conditions for the spread of epidemics.”*

### Providing information

Asylum seekers highlighted that they were unaware that the IHA was voluntary and that they could have declined to participate if they wished so. The nursing professionals underscored the significance of apprising clients about their entitled services, recognizing that clients may lack awareness regarding the appropriate avenues for seeking treatment autonomously and knowledge regarding for example infectious diseases and vaccinations or potentially traumatic experiences. They considered information provision as a tool for establishing a trust relationship between client and nurse. According to the authorities, providing information is one of the primaries aims of the IHA. However, there was no clear agreement for organizing health information sessions, whether individually or in groups. They expressed that these sessions should aim to identify the client’s illnesses, medications, and symptoms and clients could receive information about health services, their rights, and their obligations.

*“Even so, the client might have participated in the initial information, but at least the nurse at that centre will become familiar to the client, and the client will probably also receive information about the service practices at that centre. There is also an opportunity to share information about the Finnish service system, as well as the applicant’s rights regarding health services, and that is likely the central aim.”*

## Discussion

This study, to our knowledge, is the first to examine the aims of IHAs simultaneously from the perspectives of asylum seekers, nurses, and AHAs. The results can be succinctly summarized as follows: the key objectives of asylum seekers’ IHAs are a comprehensive health and needs assessment, prevention of infections, and provision of information. The primary differences, the assessment of service needs for individuals in vulnerable situations and the accessibility of information, are elaborated in the following.

Hearing asylum seekers’ perspective is a positive step towards achieving more balanced power relations. Asylum seekers’ experiences of being objectified and receiving insufficient information align with previous research [12, 13]. In a Swedish study investigating asylum seekers’ experiences with health examinations (HEs), nearly two out of five participants reported weak communication, and more than half mentioned receiving limited information about healthcare. In the present study, asylum seekers perceived the primary aim of the IHA to be infection control, but they also expressed a need for additional efforts in psychological assessments. The migrants’ experience of inadequate healthcare services [23] may stem from a failure to adequately recognize their needs. Furthermore, asylum seekers noted that they were unaware that the IHA was voluntary, highlighting a significant gap in communication and informed consent. This poses important ethical considerations that should be better considered by health professionals responsible for offering the IHA.

The expertise and practices of nurses are essential from the perspectives of both effectiveness and power relations. The findings emphasize the need to clarify nurses’ practices, particularly in providing information and assessing vulnerability. Nurses felt more consistent in performing clear health status assessments and infection prevention tasks than in intuitively assessing vulnerability. The study indicates that asylum seekers should receive information about potential symptoms and treatment options, along with reassurance that encourages them to share their concerns. Therefore, nurses should develop the skills necessary to build trusting relationships with their clients. This research suggests that reception center nurses would benefit from greater awareness of asylum seekers’ health needs, available services, and the asylum process, in addition to their basic nursing duties. While the WHO [24] has established global competency standards for health workforce providing care for refugees and migrants, there remains a need to align these standards with practical guidelines in this specific context.

Authorities responsible for issuing directives ultimately shape the resources and practices of nurses and the experiences of asylum seekers. The primary difference between the perspectives of nurses and authorities lies in their definitions of assessing vulnerability. Nurses prioritize identifying individuals within vulnerable groups as a key objective, whereas authorities primarily frame this as a special needs assessment within the broader health and needs evaluation. From the authorities’ viewpoint, infection prevention is a key aspect of the IHA, reflecting their priorities in safeguarding public health. Surprisingly, asylum seekers, in turn, expressed trust in the authorities’ handling of this matter. Notably, only the authorities identified providing information on health and eligibility for services as one of the key aims of the IHA. This lack of information has been recognized as a significant barrier to accessing care among migrants [25]. Addressing these issues is essential for improving access to care and ensuring that the IHA meets the needs of asylum seekers effectively.

### Strengths and limitations

The research was conducted in accordance with good research practices, and analyses were consistently carried out. All the research phases are reported in detail, and the interview guide, along with a summary of the findings, can be found in the supplementary materials. Through this systematic way of reporting, readers can assess credibility [26]. Purposive sampling facilitated the collection of rich data, and data saturation was achieved for all groups [27] proposed four strategies: authenticity, credibility, criticality, and integrity, to enhance rigor in qualitative description. In this study, authenticity was ensured by selecting research methods that allowed participants to express themselves freely and analyse data in a manner that honoured the diversity of participants. These methods included purposeful sampling, thematic interviews, thematic analysis, professional interpretation, and transcription, as well as providing a rich description of the data. Credibility was established by employing positional reflexivity and by considering the interviewers’ ability to gain an insider perspective. Each interviewer shared a similar background with the interviewees they engaged with. Throughout the entire study process, each decision was critically evaluated, and researchers’ possible biases were considered.

As natural for a qualitative study, this study did not aim for generalizations, and the findings may be relevant only to similar healthcare settings. All interviewers were affiliated with THL during the study, posing a potential risk that participants may have prioritized the inclusion of infection prevention as one of the aims, given that THL serves as the authority offering guidance on infection control. The interview guide was initially created for development work purposes, and the idea of combining these datasets for research emerged later. In the analysis of the interview data, nurses explicitly identified the recognition of vulnerability as one of the central aims of the IHA. Subsequently, other sections of the interview data containing questions related to the identification of vulnerability were also incorporated into the analysis. It is important to point out that asylum seekers’ interviews were set up on short notice, potentially leading to the small number of more vulnerable individuals. Another constraint was the limited time available during purposive sampling due to frequent relocations of asylum seeker participants between reception centres. The interviews were carried out in native languages and the findings and extracts are presented in English. The translation process may have compromised the authenticity. One might argue that the dataset from 2019 is outdated, but recent years, marked by the COVID-19 pandemic and the high number of Ukrainian refugees fleeing from their homes, might have influenced the conduct of the study due to variations of number and status of the applicants. The analysis could have been conducted simultaneously for the entire dataset in collaboration with two researchers and perhaps by applying computer assistance (e.g., Atlas TI). Due to data transfer restrictions this possibility was not employed. This approach might have provided a clearer overall picture of the similarities and differences, but the rich descriptions of each group may have been compromised.

## Conclusion

The present study integrates the perspectives of asylum-seeking patients, nurses as healthcare providers, and asylum health officials on the aims of asylum seekers’ Initial Health Assessments (IHAs). Based on the findings, recommendations can be made to improve the experience of asylum seekers, the practices of healthcare professionals, and the guidelines governing IHAs.

Asylum seekers require comprehensive health assessments and access to information concerning their health and rights. The working approaches and knowledge of reception center nurses should be reviewed and enhanced to meet these needs. Authorities responsible for directives and resources must clarify their instructions regarding the information provision and the assessment of vulnerability.

The findings of this study can inform the development of health information sessions and nurse training programs and help evaluate whether the intended aims are being achieved. This study offers valuable insights from both human suffering reduction and economic perspectives. An optimal initial health assessment can streamline the service pathway and asylum process, facilitating more efficient resource allocation.

## Supplementary Information

Supplementary Material 1.

Supplementary Material 2.

Supplementary Material 3.

Supplementary Material 4.

## Data Availability

Data available from the corresponding author (katri-leena.mustonen@thl.fi) on request due to privacy/ethical restrictions.

## Abbreviations

AHA: Asylum Health Authorities
EU: European Union
HE: Health Examination
IHA: Initial Health Assessment
TERTTU: Developing the Health Examination Protocol for Asylum Seekers in Finland: A National Development Project
THL: Finnish Institute for Health and Welfare
QD: Qualitative Description
UNHCR: United Nations High Commissioner for Refugees
WHO: World Health Organization
