# Supplementary material for: Seeking the aim – perspectives of asylum seekers, nurses, and authorities on the objectives of the asylum seekers’ initial health assessment: a qualitative study
Source: BMC Health Serv Res. 2024 Sep 27;24:1132. doi: 10.1186/s12913-024-11531-w (PMC11428899; doi:10.1186/s12913-024-11531-w)
Supplement: Supplementary file 1 — Supplementary Material 1. [file 12913_2024_11531_MOESM1_ESM.pdf]

APPENDIX 1: Asylum seekers' interview structure

1. Description of the phenomenon

- Measurements
- An initial health assessment was conducted
- Objectives of initial health assessment

2. How did the objective (s) of the initial health assessment you mentioned meet your objectives?

3. What aspects did you think were important to the initial health assessment? What was good about it?

4. Did you find something difficult or uncomfortable with the initial health assessment?

5. What do you think are the key issues that should always be addressed in the initial health assessment?

6. What do you think are not part of the initial health assessment?

7. Particularly vulnerable asylum seekers include tortured and traumatized people, victims of serious illnesses and mental disorders, victims of trafficking and serious violence, and sexual and gender minorities. How do you think vulnerable asylum seekers can be identified?

8. What other things do you think would make the asylum seeker vulnerable?

9. What do you consider to be the key to establishing trust with the asylum seeker and reception centre health professionals?

10. What other views do you have on the development of initial health assessment?
