# Supplementary material for: Seeking the aim – perspectives of asylum seekers, nurses, and authorities on the objectives of the asylum seekers’ initial health assessment: a qualitative study
Source: BMC Health Serv Res. 2024 Sep 27;24:1132. doi: 10.1186/s12913-024-11531-w (PMC11428899; doi:10.1186/s12913-024-11531-w)
Supplement: Supplementary file 2 — Supplementary Material 2. [file 12913_2024_11531_MOESM2_ESM.pdf]

APPENDIX 2: Reception centre health care professionals' interview structure

Theme 1: Description of the phenomenon

- What do you think are the main aims of the initial health assessment?
- Do you have an initial health assessment template in use (if yes: What kind of? Please provide)
- Have you been instructed in performing an initial health assessment? (If yes: What kind of? From whom?)
- Do you have written instructions for performing an initial health assessment? (If yes: Produced by whom? Please provide)

Theme 2: Good practices

- What are the key issues you always address during the initial health assessment?
- What kind of things should not be included in the initial health assessment?
- How do you identify those in vulnerable position?
- What kind of things facilitate building a relationship of trust with the client?
- How do you know that the initial health assessment has met the aims you mentioned earlier?

Theme 3: Challenges

- What do you think are the biggest challenges in conducting an initial health assessment?
- How do you intend to address these challenges / how do you think they could be addressed?

Theme 4: Development needs and suggestions

- What themes do you think the TERTTU initial health assessment model should include?
- How would you improve the use of existing resources in the implementation of the initial health assessment?
- What other ideas would you have for developing the initial health assessments?
- How has the TERTTU project influenced your work? Have you received enough information about it / what kind of information would you like to receive about this project?
- What kind of in-service training would you like to receive in order to be able to perform initial health assessments better than before?
