# Supplementary material for: Seeking the aim – perspectives of asylum seekers, nurses, and authorities on the objectives of the asylum seekers’ initial health assessment: a qualitative study
Source: BMC Health Serv Res. 2024 Sep 27;24:1132. doi: 10.1186/s12913-024-11531-w (PMC11428899; doi:10.1186/s12913-024-11531-w)
Supplement: Supplementary file 3 — Supplementary Material 3. [file 12913_2024_11531_MOESM3_ESM.pdf]

APPENDIX 3: Asylum health authorities’ interview structure

Theme 1: Description of the Phenomenon

-What are, in your opinion, the most important objectives of initial health assessments?
-How does the Finnish Immigration Service promote the achievement of these objectives?
-In your view, how are these objectives currently being realized?

Theme 2: Best Practices

-What are the key elements that should always be addressed in an initial health assessment?
-Are there any aspects that, in your opinion, do not belong to the scope of an initial health assessment?

Theme 3: Challenges

-According to you, what are the most significant challenges in the implementation of initial health assessments?
-How could these challenges be addressed?

Theme 4: Developmental Needs and Suggestions

-What themes should be included in the model for initial health assessment?
-What definition should be used when discussing individuals in a vulnerable position?
-What challenges exist in this definition? (The interviewer refers to the EU legislation's definition of individuals in a vulnerable position, such as those who have experienced torture and trauma, suffer from serious illnesses and mental health disorders, victims of human trafficking and serious violence, as well as sexual or gender minorities.)
-Do you have any other perspectives related to the development of initial health assessments?
