# Supplementary material for: Seeking the aim – perspectives of asylum seekers, nurses, and authorities on the objectives of the asylum seekers’ initial health assessment: a qualitative study
Source: BMC Health Serv Res. 2024 Sep 27;24:1132. doi: 10.1186/s12913-024-11531-w (PMC11428899; doi:10.1186/s12913-024-11531-w)
Supplement: Supplementary file 4 — Supplementary Material 4. [file 12913_2024_11531_MOESM4_ESM.pdf]

Table 1 Summary of the findings

	Themes	Categories	Subcategories
**Asylum seekers**	Survey of the need for health services<br>Infection control	Healthy<br>Sick	
**Reception centre nurses**	Assessment of treatment needs	Health status<br>Follow up care	• Interview<br>• Health examination<br>• Doctor's appointment
	Preventing infections	Identifying<br>Treatment<br>Containment	• Interview<br>• Screening<br>• Vaccinations
	Identifying vulnerable persons	Identification<br>Follow up<br>Asylum process	• Interview
**Asylum health authorities**	Providing information	Health services<br>Rights & obligations	• Individual<br>• Group
	Comprehensive health and special needs assessment	Timeline<br>Statutory	• Somatic health<br>• Psychosocial wellbeing<br>• Special needs
	Preventing infections	Individual<br>Public	• Screening<br>• Vaccinations<br>• Symptoms
